# The transcriptomic response to irinotecan in colon carcinoma bearing mice preconditioned by fasting

**DOI:** 10.18632/oncotarget.26776

**Published:** 2019-03-15

**Authors:** Franny Jongbloed, Sander A. Huisman, Harry van Steeg, Jeroen L.A. Pennings, Jan N.M. IJzermans, Martijn E.T. Dollé, Ron W.F. de Bruin

**Affiliations:** ^1^ Department of Surgery, Erasmus University Medical Center, Rotterdam, The Netherlands; ^2^ Laboratory for Health Protection Research, National Institute of Public Health and The Environment, Bilthoven, The Netherlands; ^3^ Department of Human Genetics, Leiden University Medical Center, Leiden, The Netherlands

**Keywords:** fasting, colorectal carcinoma, irinotecan, transcriptome analysis, stress resistance

## Abstract

**Background:**

Irinotecan use is limited due to severe toxicity. Preconditioning by fasting (PBF) protects against side effects of irinotecan while preserving its antitumor activity. The mechanisms underlying the effects of PBF still need to be elucidated. Here, we investigated the transcriptional responses of PBF on irinotecan in both tumor and healthy liver tissue.

**Experimental approach:**

Male BALB/c mice were subcutaneously injected with C26 colon carcinoma cells. Twelve days after tumor inoculation, two groups were fasted for three days and two groups were allowed food ad libitum (AL). Subsequently, both groups received one dose of irinotecan. Twelve hours after administration mice were sacrificed and blood, tumor and liver tissue were harvested. Blood samples were analyzed to determine liver, kidney and bone marrow function, tissues were used for transcriptome analyses.

**Key results:**

The AL irinotecan group showed worsened organ function and decreased leukocyte numbers. These effects were abated in PBF animals. PBF led to an altered transcriptional response in the liver of irinotecan-treated mice, including decreased cellular injury and increased stress resistance. Hepatic metabolism of irinotecan was also significantly changed due to PBF. The transcriptional response of tumor tissue observed after PBF was hardly affected compared to AL fed animals.

**Conclusions:**

Transcriptional changes after PBF to irinotecan treatment showed an improved protective stress response in healthy liver but not in tumor tissue, including changes in irinotecan metabolism. These data help to unravel the mechanisms underlying the effects of fasting on irinotecan and help to improve outcome of chemotherapeutic treatment in cancer patients.

## INTRODUCTION

Colorectal cancer (CRC) is the second most diagnosed cancer in women and the third most diagnosed in men. Estimated new colorectal cancer cases account for 1.4 million cases and 693,900 deaths worldwide occurring in 2012 [1]. At initial presentation, 15–20% of patients already have liver metastases and another 45% is diagnosed with liver metastases in the follow-up after resection of the primary tumor [2]. Irinotecan is a pro-drug of the topoisomerase-I inhibitor SN-38, and is applied in first and second line chemotherapy treatment for colorectal carcinoma [3, 4]. However, irinotecan can induce severe and unpredictable side effects including myelosuppression, diarrhea, and in some cases even death as a complication of side effects [5].

Dietary restriction (DR) is a method to trigger highly conserved survival mechanisms that enhance the resistance of organisms against stressors and diseases [6]. We recently showed that three days of fasting prior to a toxic dose of irinotecan significantly prevented the occurrence of side effects in *Apc*-mutant mice [7]. Furthermore, tumor size and proliferation were reduced equally in *ad libitum* (AL) fed and fasted animals. Levels of the active metabolite of irinotecan, SN-38, were significantly lower in plasma and liver tissue from fasted mice, indicating that dietary preconditioning was able to reduce the systemic toxicity of SN-38 while the phenotypical effect on tumor tissue remained unaltered [7, 8]. These data support the concept of differential stress resistance (DSS), which states that tumor cells are unable to elicit a protective response since they remain driven towards growth due to mutations in onco- and tumor suppressor genes [8, 9]. However, the molecular mechanisms that govern these processes remain largely unknown.

Insight into these mechanisms could facilitate translational research into clinical practice since it may reveal alternative approaches to DR such as specialized forms of DR or DR-mimetics which may induce similar effects without the disadvantages of fasting, including additional body weight loss in cancer patients. Therefore, in this study we investigated the transcriptional responses to preconditioning by fasting (PBF) and irinotecan exposure in tumor and in healthy liver tissue.

## RESULTS

### Fasting induces a chemoprotective phenotype in the liver

Male BALB/c mice with subcutaneous C26 colon carcinoma tumors were divided over a group fed *ad libitum* (AL) and a group with preconditioning by fasting (PBF) for three days, after which both groups received irinotecan intraperitoneally. All mice were subsequently fasted for two hours followed by AL access to food for 10 hours, until the moment of sacrifice. The mice in the AL irinotecan group consumed on average 2.9 grams of chow per mouse in this period. The PBF irinotecan group had average intake of 3.5 grams per mouse (data not shown).

**Figure 1:**
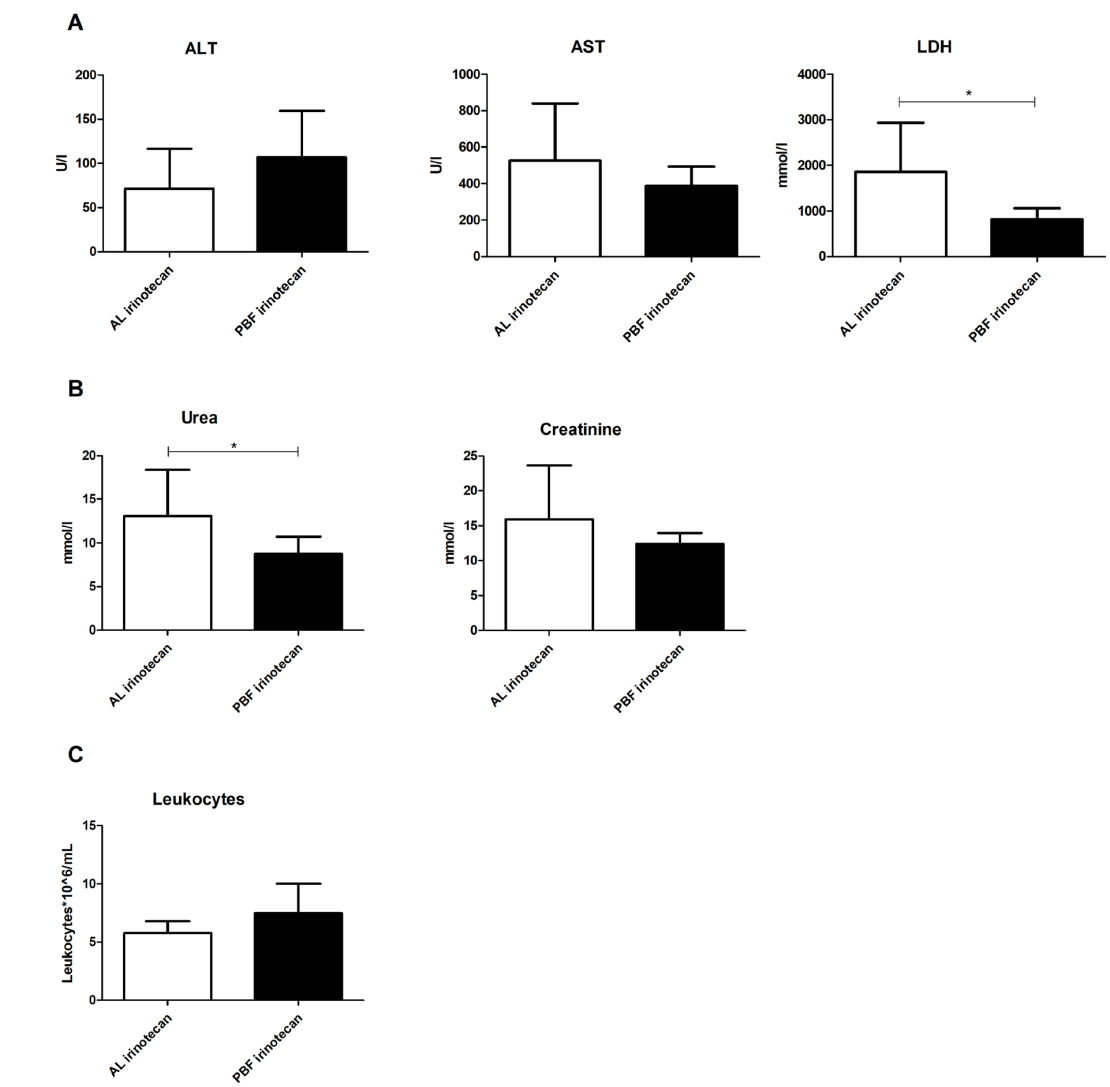
Serum markers 12 hours after irinotecan administration to ad libitum (AL) or preconditioned by fasting (PBF) mice (**A**) Markers of liver function: aspartate transaminase (AST), alanine transaminase (ALT) and lactate dehydrogenase (LDH). (**B**) Markers of kidney function: urea and creatinine. (**C**) Leukocyte number. ^*^=*P* < 0.05, ^**^=*P* < 0.01.

Induction of toxicity by irinotecan and possible protective effects of PBF were determined via markers of general (LDH) and liver specific (ALT and AST) cellular injury, and kidney function (urea and creatinine). Serum measurements of these markers are known to increase upon cell damage and cell death or kidney dysfunction. Leukocyte depletion is a common side effect of irinotecan treatment and therefore, leukocyte numbers were used as markers of bone marrow toxicity. Administration of irinotecan in mice fed AL led to liver and kidney injury as seen by high levels of AST, LDH (Figure 1A), and urea (Figure 1B). In the PBF irinotecan group these markers were on average affected to lesser extent than in the AL irinotecan group and both LDH and urea levels were significantly lower in the PBF irinotecan compared to the AL irinotecan group. Irinotecan caused a depletion of leukocytes in AL fed mice, but not in PBF animals (Figure 1C). Collectively, these results confirm that fasting induces a systemic chemo-protective phenotype [10].

### Liver transcriptome analysis

To investigate the chemo-protective phenotype induced by PBF after irinotecan exposure at the molecular level in healthy and tumor tissue, the transcriptomes of liver and tumor tissue were directly compared between irinotecan exposed AL and PBF mice.

### Principal component analysis

**Figure 2:**
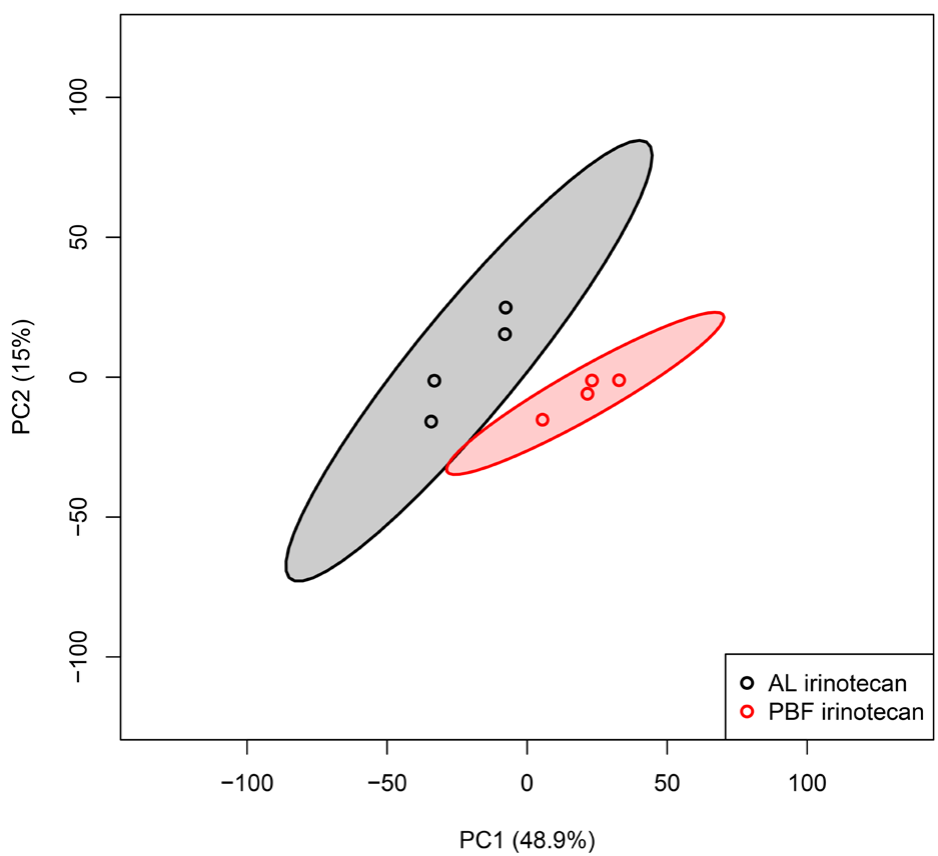
Unbiased principal component analyses (PCA) of liver samples, based on all probe sets in the microarray Principal component (PC) 1 is depicted on the x-axis and PC2 is depicted on the y-axis, including the percentage of variance explained by each PC. Each symbol represents one sample of one mouse. Samples of the same group are shown in the same color. Colored field represents 95% confidence intervals of the group with the circles in the same color. Abbreviations: AL, ad libitum; PBF, preconditioned by fasting.

To investigate variability between the irinotecan-treated groups, we performed an unbiased principal component analysis (PCA) including all probe sets in the microarray of liver tissue, including 95% confidence intervals (CI). When comparing liver tissue of AL irinotecan and PBF irinotecan, 48.9% of the variance was explained by principal component (PC) 1 and 15.0% by PC2 (Figure 2). A pattern of distinct clustering of the two groups was seen. The PBF irinotecan group had a smaller CI than de AL irinotecan group, and the groups had no overlap. These data point towards a differently regulated and homogeneous response to irinotecan in the liver due to PBF.

### Expression and pathway analyses

To compare expression profiles between the different experimental groups, the numbers of differentially expressed probe sets (DEPS) were calculated. In the liver, 3,636 DEPS were found between the AL irinotecan and the PBF irinotecan groups, with 1,554 DEPS downregulated and 2,082 upregulated. To explore canonical pathways enriched by PBF, the 3,636 DEPS resulting from the comparison AL irinotecan versus PBF irinotecan were analyzed. A total of 41 pathways were found to be regulated, defined by a significance of *P* < 0.05 and a z-score of >1 or <1 (Table 1). Of these 41 pathways, 32 were activated and nine were inhibited. Upregulated pathways were mainly involved in in biosynthesis of fatty acids, lipids and hormones. Another class overrepresented was amino acid degradation, mainly (iso)leucine, valine and tryptophan degradation pathways. Two other pathways involved in oxidative stress and injury, glutathione-mediated reactions and NRF-mediated stress response, were upregulated as well. Analyses of the downregulated pathways classified these mainly as response to injury and cellular proliferation as well as cytokine signaling. Xenobiotic metabolism appeared reduced as indicated by inhibition of the aryl hydrocarbon receptor (AHR) pathway. Analysis of upstream transcription factors (TFs) revealed activated TFs involved in stress resistance, including NRF1, NRF2 and PPARg, as well as upregulation of metabolic TFs, including SREBF1 and SREBF2 (Supplementary Table 1). Of the downregulated TFs, GATA binding proteins were mainly represented as well as proto-oncogenes including MYB and ETS1.

**Table 1:** Overview of the top overrepresented canonical pathways in liver ranked by their z-score Liver – AL irinotecan vs. Fasted irinotecan

Canonical pathway	Pathway classification	*P*-value	Genes ratio	Z-score
**Superpathway of Cholesterol Biosynthesis**	Fatty acids and lipids biosynthesis	3.14^E^-02	19/28 (67.9%)	**+4.359**
**tRNA Charging**	Aminoacyl-tRNA charging; biosynthesis	2.41^E^-06	16/39 (41.0%)	**+4.000**
**Glutathione-mediated Detoxification**	Detoxification	4.18^E^-04	11/31 (35.5%)	**+3.317**
**Cholesterol Biosynthesis I/II/III**	Fatty acids and lipids biosynthesis	7.98^E^-08	10/13 (76.9%)	**+3.162**
**NRF2-mediated Oxidative Stress Response**	Cellular stress and injury	8.06^E^-10	53/193 (27.5%)	**+3.138**
**Mevalonate Pathway I**	Fatty acids and lipids biosynthesis	1.57^E^-06	9/13 (69.2%)	**+3.000**
**Superpathway of Geranylgeranyldiphosphate Biosynthesis I**	Biosynthesis	3.47^E^-05	9/17 (52.9%)	**+3.000**
**Glutathione Redox Reactions I**	Biosynthesis	4.00^E^-03	8/24 (33.3%)	**+2.828**
**Noradrenaline and Adrenaline Degradation**	Hormones Degradation	1.29^E^-02	10/40 (25.0%)	**+2.530**
**Leucine Degradation I**	Amino acid degradation	1.40^E^-04	6/9 (66.7%)	**+2.449**
**Ethanol Degradation II**	Alcohols Degradation	2.13^E^-02	9/37 (24.3%)	**+2.333**
**Estrogen Biosynthesis**	Hormones Biosynthesis	2.57^E^-05	15/41 (36.6%)	**+2.324**
**Fatty Acid B-oxidation I**	Fatty acid and lipids degradation	1.25^E^-04	12/32 (37.5%)	**+2.309**
**Stearate Biosynthesis I**	Fatty acid and lipids biosynthesis	3.16^E^-03	12/44 (27.3%)	**+2.309**
**Ketogenesis**	Metabolites and Energy	3.03^E^-03	5/10 (50.0%)	**+2.236**
**Colanic Acid Building Blocks Biosynthesis**	Cell Structures Biosynthesis	1.62^E^-02	5/14 (35.7%)	**+2.236**
**Zymosterol Biosynthesis**	Fatty acids and lipids biosynthesis	2.14^E^-03	4/6 (66.7%)	**+2.000**
**Isoleucine Degradation I**	Amino Acids Degradation	4.27^E^-04	7/14 (50.0%)	+1.890
**Glutaryl-CoA Degradation**	Carboxylates Degradation	1.16^E^-03	7/16 (43.8%)	+1.890
**Valine Degradation I**	Amino Acids Degradation	2.61^E^-03	7/18 (38.9%)	+1.890
**Tryptophan Degradation X/III**	Amino Acids Degradation	1.93^E^-02	7/25 (28.0%)	+1.890
**Ethanol Degradation IV**	Alcohols Degradation	1.93^E^-02	7/25 (28.0%)	+1.890
**Androgen Signaling**	Nuclear receptor Signaling	3.91^E^-02	23/137 (16.8%)	+1.667
**Aldosterone Signaling in Epithelial Cells**	Nuclear Receptor Signaling	4.42^E^-02	27/168 (16.1%)	+1.667
**Serotonin Degradation**	Hormones Degradation	1.29^E^-02	16/77 (20.8%)	+1.500
**Retinoate Biosynthesis I**	Vitamins Biosynthesis	3.52^E^-02	8/34 (23.5%)	+1.414
**Heme Biosynthesis I**	Heme Biosynthesis	1.67^E^-03	5/9 (55.6%)	+1.342
**Dolichyl-diphosphooligosaccharide Biosynthesis**	Carbohydrates Biosynthesis	5.03^E^-03	5/11 (45.5%)	+1.342
**Phenylalanine Degradation IV**	Amino Acids Degradation	1.62^E^-02	5/14 (35.7%)	+1.342
**y-linolenate Biosynthesis II**	Fatty acids and lipids biosynthesis	3.76^E^-02	5/17 (29.4%)	+1.342
**14-3-3-mediated Signaling**	Cell Cycle Regulation; Apoptosis	3.57^E^-04	29/131 (22.1%)	+1.213
**Putrescine Degradation III**	Amines and Polyamines Degradation	1.20^E^-02	7/23 (30.4%)	+1.134
**Intrinsic Prothrombin Activation Pathway**	Cellular Stress and Injury; cardiovascular signaling	6.46^E^-03	11/42 (26.2%)	-2.333
**Osteoarthritis Pathway**	Cellular Stress and Injury	2.73^E^-02	34/212 (16.0%)	-2.117
**Aryl Hydrocarbon Receptor**	Cell Cycle Regulation; Apoptosis; Xenobiotic Metabolism; Nuclear Receptor Signaling	2.59^E^-04	31/141 (22.0%)	-2.111
**STAT3 Pathway**	Cellular growth and proliferation and development	1.25^E^-03	22/97 (22.7%)	-1.706
**LPS-IL-1 Mediated Inhibition of RXR Function**	Nuclear Receptor Signaling	4.57^E^-06	49/222 (22.1%)	-1.604
**Coagulation System**	Cellular Stress and Injury	7.38^E^-05	13/35 (37.1%)	-1.387
**Inhibition of Angiogenesis by TSP-1**	Cardiovascular Signaling	3.52^E^-02	8/34 (23.5%)	-1.342
**HIPPO Signaling**	Organismal Growth and Development	9.08^E^-03	18/87 (20.7%)	-1.265
**Acute Phase Signaling**	Cytokine Signaling	1.41^E^-07	44/170 (25.9%)	-1.219

All canonical pathways with a z-score of <−1.000 or >+1.000 are listed. Pathways with a significant z-score of ≤−2.000 or ≥+2.000 are depicted in bold. Genes ratio= the number and percentage of genes differentially expressed in ratio to the total number of genes involved in the pathway.

### Tumor transcriptome analysis

#### Principal component analysis

**Figure 3:**
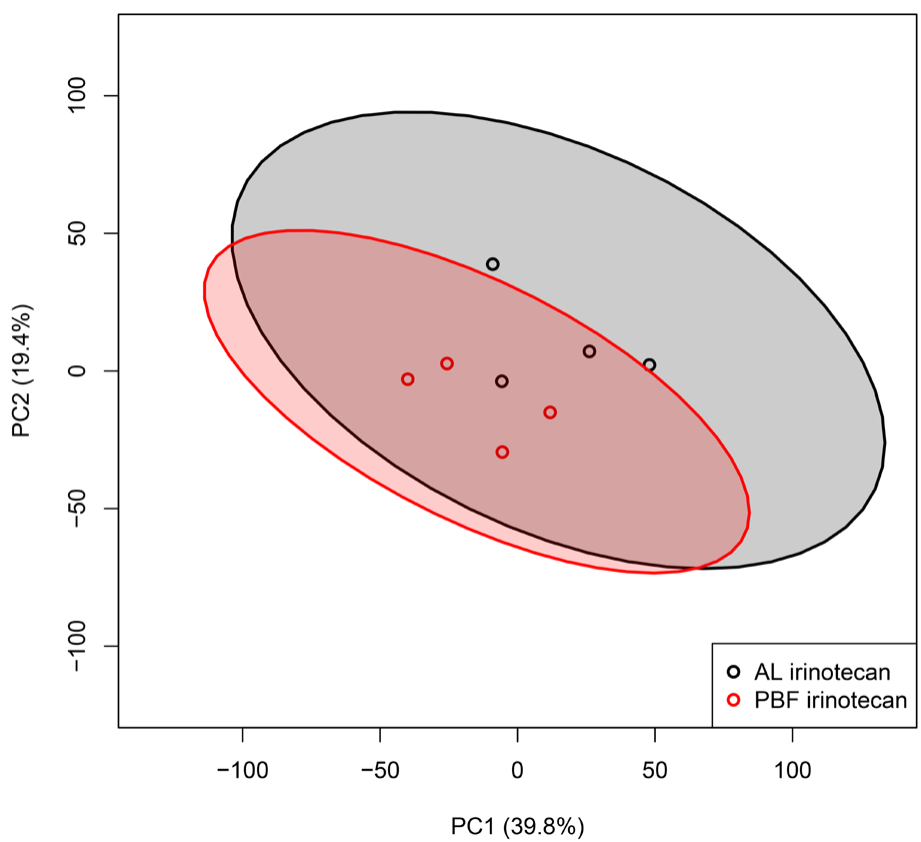
Transcriptome analysis of tumor tissue Unbiased principal component analyses (PCA) of tumor samples, based on all probe sets in the microarray. Principal component (PC) 1 is depicted on the x-axis and PC2 is depicted on the y-axis, including the percentage of variance explained by each PC. Each symbol represents one sample of one mouse. Samples of the same group are shown in the same color. Colored field represents 95% confidence intervals of the group with the circles in the same color. Abbreviations: AL, ad libitum; PBF, preconditioned by fasting.

Next, the transcriptomes of the tumor samples were analyzed using a similar approach as for the liver samples. With a total of 39.8% of the variance explained by PC1 and 19.4% by PC2, the PCA plot of the tumor tissue of the two experimental groups showed high heterogeneity among the groups with large intragroup variability (Figure 3). The AL and PBF irinotecan groups were not clearly separated as clusters as shown by the overlapping 95% CIs. These PCA plots show a heterogeneous response of tumor tissue treated with irinotecan, and this response was not altered by three days of PBF.

#### Expression and pathway analyses

Calculation of the number of DEPS in the tumor tissue was done similarly as in the liver. One hundred and sixty DEPS were found between the AL irinotecan and the PBF irinotecan groups with 95 DEPS downregulated and 65 upregulated. Pathway analysis was performed of the 160 DEPS differentially regulated between AL irinotecan and fasting irinotecan in tumor tissue. This analysis revealed no enriched pathways, which suggests that fasting had no significant effect on the transcriptome of tumor tissue. Since no pathways were differentially regulated, analysis of upstream transcription was not performed.

#### Irinotecan metabolism related gene expression profiles in liver and tumor tissue

**Figure 4:**
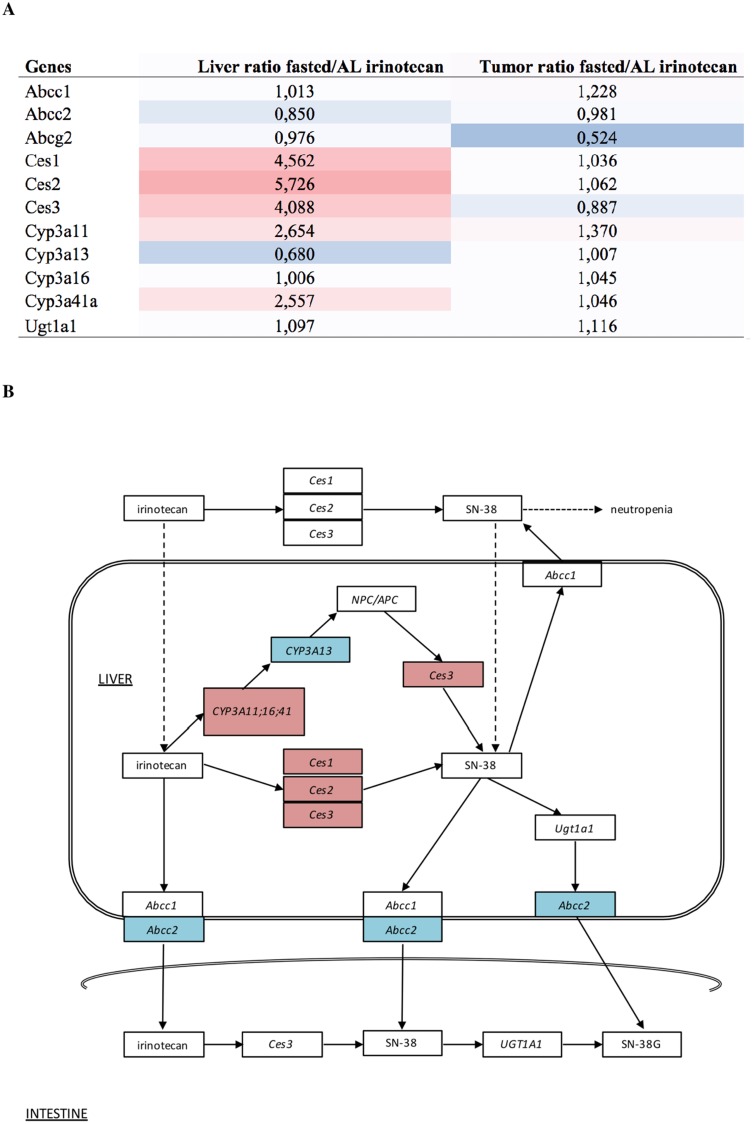
(**A**) Heatmap and expression values of probe sets related to irinotecan metabolism in both liver and tumor. Expression levels are depicted as the ratio of levels of fasted divided by ad libitum fed mice. The coloring scale represents the degree of either upregulated (red) or downregulated (blue) expression values. (**B**) Overview of the pathway of irinotecan metabolism outside and inside the liver cell, including the transportation into the intestine. The coloring scale represents the expression values in the liver depicted as the ratio of levels of fasted divided by ad libitum fed mice. These levels are also depicted in figure A. Dashed arrows are suggested actions of which the exact mechanisms are unclear. Abbreviations: AL, ad libitum; Ces, carboxylesterase; SN-38, active metabolite of irinotecan; SN-38G, SN-38 glucuronide form; Abcc, ATP binding cassette subfamily C; NPC/APC, carbonyloxycamptothecin; Cyp3a, cytochrome P450 3A; Ugt1a1, Uridine glucuronosyltransferase 1A1.

We have previously shown that both irinotecan and its active metabolite SN-38 levels are lower in the livers and in plasma of PBF mice, compared to AL fed mice [10]. Here, we examined specifically expression differences of genes coding for proteins involved in the metabolism and transportation of irinotecan, using the AL irinotecan group as a reference (Figure 4A). In the liver, mRNA expression of *Ces1, Ces2* and *Ces3* was upregulated by PBF. Of the alternative conversion pathway to SN-38, via cytochrome P450 (*Cyp*)*3a*11, *Cyp3a41, Cyp3a13* and *Ces3*, the expression levels of *Cyp3a11, Cyp3a41* and *Ces3* were upregulated whereas *Cyp3a13* was downregulated. The ATP binding cassette subfamilies (*Abc)* C and *Abcg*, involved in the transport of SN-38 out of the cell, showed lower expression after PBF compared to AL irinotecan-treated liver tissue (Figure 4B). *Ugt1a1*, also involved in the transport of SN-38 out of the cell as well as conversion of SN-38 to the inactive metabolite SN-38G, was not differentially regulated. The expression levels of these genes were not or hardly affected by PBF in tumor tissue, with the exception of *Abcg2* which was downregulated due to PBF (Figure 4A). These data suggest a higher conversion rate of irinotecan to SN-38 due to PBF, while the transport out of the cell appears inhibited. In tumor tissue, the same genes were not or only marginally affected due to fasting.

## DISCUSSION

In this study, we confirm that PBF prior to treatment with irinotecan is able to reduce liver toxicity as well as bone marrow depression in mice carrying subcutaneous colorectal carcinoma. Previously, we showed that these signs of somatic resistance to irinotecan were accompanied by a reduction in other side effects as body weight loss and diarrhea without compromising the antitumor effect [7, 10]. Here we set out to retrieve mechanistic insights in the chemoprotective effects of BPF through transcriptomic analyses of healthy liver and tumor tissue after treatment with irinotecan.

Since the most prominent effect of PBF is the reduction of toxic side effects of chemotherapy, its effects should be sought primarily in healthy tissue including the liver in which the majority of its drug metabolism takes place. In this study, PCA showed that irinotecan administration after PBF led to a more homogeneous gene expression profile compared to AL, with a smaller confidence interval in healthy liver tissue. Pathway analysis in liver tissue based on expression differences between irinotecan exposed AL and PBF mice, revealed insights into the underlying mechanisms of the PBF-induced protective phenotype. Firstly, response to injury and cytokine signaling were both reduced and NRF2-mediated oxidative stress response was highly activated in the PBF mice. Since cytokines are crucial players in the activation and adaptation to oxidative stress, this reduction is evidence of increased stress resistance induced by PBF and may also account for the reduction in DNA damage in healthy tissues after chemotherapy [11]. The activation of NRF2 is strongly associated with the beneficial effects of DR [12, 13], and might precondition healthy cells to become protected against the damage-induced response of irinotecan [14–16]. Posttranscriptional validation is needed to further understand the exact role of these pathways on stress resistance and prevention of side effects of chemotherapy. Secondly, fatty acid metabolism was activated as evidenced by cholesterol, mevalonate and fatty acid oxidation pathways. Previous data showed downregulation of these pathways due to fasting alone [12, 17]. In our study, mice were allowed to eat ad libitum during ten hours after irinotecan treatment awaiting sacrifice to obtain tissues and blood 12 hours after irinotecan administration. The transition from a fasted to a fed state during this period may well have caused the upregulation of metabolism. Nevertheless, this transitional phase could be a contributing factor to the protective phenotype as fatty acid metabolism has been linked to resistance against the adverse effects of chemotherapy and resistance to SN-38 in particular via upregulation of transcription factor SREBF [18, 19]. Indeed, SREBF1 and SREBF2 were amongst the highest predicted activated transcription factors in our analysis. Again, further studies could focus on these metabolic factors and their link to improved chemotherapeutic resistance due to PBF.

The changes induced by PBF in healthy liver tissue were absent in the tumor transcriptome analysis. The PCA plots of the AL and PBF groups revealed a high heterogeneity within the tumor tissue, which is in contrast with the orchestrated response of the liver. The low number of DEPS revealed no enriched pathways due to PBF in tumors of irinotecan-treated mice. Therefore, no upstream TFs were found as well. These findings add to the evidence that PBF does not negatively affect the antitumor effect of irinotecan on tumor tissue, which is in line with our previous results [7, 10].

Previously, we have found decreased levels of the active metabolite of irinotecan, SN-38, in plasma as well as liver but not in tumor tissue of fasted mice [10]. Carboxylesterases (Ces) one to three are needed to convert irinotecan in to its active metabolite SN-38 [20]. The relative upregulation of the carboxylesterases in fasted liver tissue in this study would suggest an increased hydrolysis of irinotecan into SN-38 [7, 21]. However, Cyp3a13, which is also able to convert irinotecan into SN-38, was downregulated which may reduce the total amount of SN-38 produced. Downregulation of the ABC transporter ABCC2 is indicative of decreased transport of SN-38 out of the cell [22–24]. In addition, pathway analysis revealed activation of the glutathione system. Glutathione is associated with cellular protection, and the increased expression of glutathione as seen in our analysis is in line with the decreased liver toxicity as observed in this study. We hypothesize that PBF alters the metabolism of irinotecan and protects healthy cells from its toxicity, while preserving its antitumor efficacy. In an earlier study, Huisman *et al*. found no significant differences in mRNA levels of Ces2 between AL and fasted groups in both liver and tumor tissue, while SN-38 levels were lowered in both liver and plasma [10]. However, in this study CES2 transcription was measured on different time points (i.e. one, eight and 12 hours after irinotecan injection), while we only have expression levels at ten hours after PBF which complicates a direct comparison. In contrast to liver tissue, changes in expression levels of enzymes involved in irinotecan metabolism in tumor tissue after PBF were far less prominent underscoring that PBF does not abrogate antitumor activity.

Since pharmacokinetic studies showed that the half-life of SN-38 is approximately 12 hours [10], we choose to explore the transcriptional changes at this time point, using one dosage of irinotecan in the therapeutic window in which the effects of both irinotecan and PBF would be maximal. Analyses of multiple time points and with different dosages of irinotecan would likely improve our understanding of the results we obtained. Finally, this study only partially explains how PBF changes irinotecan metabolism and how it protects the organism, but not the tumor, against irinotecan exposure. Addressing the impact of PBF on a posttranscriptional level will likely improve our understanding of the mechanisms of PBF-induced chemoprotection.

In conclusion, we show that three days of PBF results in protection against the side effects of irinotecan treatment and activates a protective stress response in healthy liver tissue. These effects were absent in tumor tissue, which explains why it does not abrogate its antitumor efficacy. These results further strengthen the oncologic safety of DR in cancer patients, where preconditioning by short-term fasting could improve both efficacy of treatment and quality of life in patients with colorectal cancer treated with irinotecan.

## MATERIALS AND METHODS

### Animals

Male BALB/c mice of 6–8 weeks old, weighing approximately 25 grams, were obtained from Charles River, Maastricht, The Netherlands. Upon arrival, animals were housed at random in individually ventilated cages (n=4 animals per cage) in a licensed biomedical facility at Erasmus University Medical Center, Rotterdam, The Netherlands. Standard laboratory conditions were maintained, i.e. temperature ∼22° C, humidity ~50%, and a 12 h light/12 h dark cycle. All mice had free access to water and food (Special Diet Services, Witham, UK) unless mentioned otherwise. Animals were allowed to acclimatize for one week before the start of the experiments. The experimental protocol was approved by the Animal Experiments Committee under the Dutch National Experiments on Animals Act, and complied with the ARRIVE and BJP guidelines [25].

### C26 colon carcinoma cells

The murine colon carcinoma cell line C26, originally derived from the BALB/c mouse was cultured in Dulbecco's Modified Eagle's Medium (Sigma Aldrich, St. Louis, MO, USA), supplemented with 10% fetal calf serum (Lonza, Verviers, Belgium), penicillin (100 units/ml) and streptomycin (100 units/ml) (Invitrogen, Auckland, New Zealand) at 37 degrees Celsius in a 5% carbon dioxide environment. Cells were harvested by brief trypsinization (0.05% trypsin in 0.02% ethylenediamine tetra-acetic acid (EDTA)). For subcutaneous injection, cells were harvested and after centrifugation, single-cell suspensions were prepared in phosphate buffered saline (PBS) to a final concentration of 2.5 × 10^5^ cells/100 μL. Cell viability was determined by trypan blue staining, and was always ≥90%.

### Experimental setup

The exact group size for each experimental group (*n* = 6/group) was equal by design. The mice were randomly divided into two groups. Since food had to be given daily to the AL fed groups, blinding of the operators to the dietary intervention groups was not feasible to execute. Experimental setup is depicted in Supplementary Figure 1.

All mice (*n* = 12) were anaesthetized (isoflurane inhalation, 5% isoflurane inhalation initially and then 2% isoflurane with a 1:1 air: oxygen mixture for maintenance of anesthesia) (Supplementary Figure 1). Both flanks were shaved for precise injection. 2.5 × 10^5^ C26 cells were injected subcutaneously on both sides in a volume of 100 μL, using a 21G needle. Tumors were allowed to grow for 12 days before start of the experiment. Mice were weighed and tumors were measured daily with digital calipers. As stated above, the mice were randomly divided into two groups (*n* = 6/group). One group was preconditioned by fasting (PBF) for three days and one group was fed AL. Subsequently, both groups were treated with a single weight-adjusted dose of 133 mg/kg (±3.3 mg/kg, and ±2.7 mg/kg respectively) of irinotecan intraperitoneally. Two hours after injection, both groups received unlimited access to food for 10 hours until the moment of sacrifice. The difference between starting amount and remaining amount of food was used to calculate food consumption per mouse for this 10-hour period. Subsequently, 12 hours after irinotecan administration, the mice were sacrificed by exsanguination.

### PBF protocol

Mice in the AL fed group were allowed unrestricted access to food, and the amount of food consumed per cage was measured daily. Before the start of the PBF period, all mice were transferred to a clean cage and mice in the PBF groups were withheld food for three days starting at 4:00 PM on a Friday until 10:00 AM on a Monday. All animals were given continuous access to water.

### Chemotherapy

Irinotecan, HCl-trihydrate 20 mg/mL (Hospira, Benelux) was used for *in vivo* experiments. Irinotecan was diluted in sodium chloride 0.9% (Braun, Melsungen, Germany) to a final volume of 200 μL per injection, and was given intraperitoneally.

### Plasma measurements of organ function

Mice were killed by exsanguination with cardiac puncture under anesthesia (isoflurane inhalation, 5% isoflurane inhalation initially and then 2% isoflurane with a 1:1 air: oxygen mixture for maintenance of anesthesia). After cardiac puncture, ± 900 μL of blood per mouse was transferred directly into 1 mL tubes (MiniCollect, Greiner Bio-one), containing EDTA. Samples were directly centrifuged (3,500 rpm; 10 min) after which the plasma was transferred to a separate tube. Plasma aspartate transaminase (AST), alanine transaminase (ALT), lactate dehydrogenase (LDH), urea, and creatinine levels were analyzed at the Central Clinical Chemical Laboratory of the Erasmus University Medical Center. Fifty μL of blood was used to measure the number of leukocytes with a Z-series Coulter Counter (Beckman Coulter, Woerden, The Netherlands).

### Tissue sampling

Liver and tumors were collected and weighed. The median liver lobe was isolated for array analysis and directly stored in RNA*later*^®^ Solution (Life Technologies Europe BV, Bleijswijk, The Netherlands) and stored at 4° C until further analysis. Viable tumor samples were identified at the border of the tumor tissue excluding necrotic parts as well as normal tissue. Only plain tumor tissues remained, and these samples were directly stored in RNA*later*^®^ until further analysis.

### RNA isolation

Plain tumor and liver samples obtained were kept at 4° C in RNA*later*^®^ Solution until further analyses. RNA isolation took place between 24 hours and 96 hours after sample collection. Total RNA was extracted via the QIAzol lysis Reagent and miRNAeasy Mini Kits (QIAGEN, Hilden, Germany), according to Qiagen protocol. Concentrated buffers RPE and RWT (QIAGEN) for washing of membrane-bound RNA and purification were added mechanically by using the QIAcube (QIAGEN, Hilden, Germany) via the miRNeasy program. Subsequently, isolated RNA was stored at −80° C. RNA concentrations were measured using the Nanodrop (Thermo Fisher Scientific™, Breda, The Netherlands) and RNA quality was assessed using the 2100 Bio-Analyzer (Agilent Technologies, Amstelveen, The Netherlands), according to manufacturer's instructions. The RNA quality was quantitatively expressed as the RNA Integrity Number (RIN, range 0–10). Out of the six tumor samples and six liver samples per group, the four samples with the highest RIN were used for microarray analyses. RIN-values of the tumor samples ranged between 7.8 and 10, the RIN-values of the liver samples ranged between 7.6 and 8.6.

### Array analysis

Microarray hybridization was done at the Microarray Department of the University of Amsterdam (The Netherlands) to Affymetrix HT MG-430 PM Array Plates, according to the Affymetrix protocols. For each group, 4 biological replicates were used. The output of the hybridization contained raw mean expression data put into CEL files. Subsequent quality control and normalization were done using the pipeline at the www.arrayanalysis.org website (Maastricht University, The Netherlands) [26]. Normalization was performed via the Robust Multichip Average algorithm, and the output of the normalization consisted of 45141 probes [27]. Both raw and normalized microarray data and their MIAME compliant metadata were deposited at the Gene Expression Omnibus database, with number GSE72484 (www.ncbi.nlm.nih.gov/geo).

### Statistical analyses

For each set of parameters means and standard errors of the mean were computed. All standard statistical tests were performed using SPSS version 21 for Windows software (Statistical Package for Social Sciences, Chicago, IL, USA) and GraphPad Prism (GraphPad Software Inc., version 5.01). Comparison of plasma markers was done using the Mann-Whitney *U*-test for non-parametric data. A *P*-value < 0.05 was considered to be significant. Microarray analyses were performed using the free software package R (R foundation). Gene expression profiles were compared using the Linear Models for Microarray Data (limma) method with correction for multiple testing using the false discovery rate (FDR) according to Benjamini and Hochberg [28]. Fold changes were expressed as the geometric mean per diet group against the corresponding AL fed control group, and cut-off values for a significant difference were put at FDR <5%. Functional annotation and analyses were performed using the Ingenuity software (http://www.ingenuity.com/products/ipa). Inhibition or activation prediction of the pathway analysis and upstream transcription regulators was predicted with Ingenuity software by calculating statistical z-scores based on the observed gene expression changes in our dataset. Via z-scores, the chance of significant prediction based on random data is reduced (http://pages.ingenuity.com/rs/ingenuity/images/0812%20upstream_regulator_analysis_whitepaper.pdf). Cut-off values for a significant activation or inhibition were put at a z-score of ≥2 or ≤-2, respectively.

## SUPPLEMENTARY MATERIALS FIGURES AND TABLES



## Abbreviations

ABC: ATP binding cassette subfamilies
AHR: aryl hydrocarbon receptor
AL: ad libitum
ALT: alanine transaminase
AST: aspartate transaminase
CES: carboxylesterase
CI: confidence intervals
CRC: colorectal carcinoma
CYP: cytochrome P450
DEPS: differentially expressed probe sets
DR: dietary restriction
DSS: differential stress resistance
EDTA: ethylenediamine tetra-acetic acid
ETS1: ETS proto-oncogene 1
FDR: false discovery rate
LDH: lactate dehydrogenase
MYB: MYB Proto-oncogene
NRF: Nuclear receptor factor
PBF: Preconditioning by fasting
PBS: phosphate buffered saline
PC: principal component
PCA: principal component analysis
PPARg: Peroxisome proliferator-activated receptor gamma
RIN: RNA Integrity Number
SREBF: Sterol regulatory element-binding protein
TF: Transcription factor
