# Supplementary material for: The transcriptomic response to irinotecan in colon carcinoma bearing mice preconditioned by fasting
Source: Oncotarget. 2019 Mar 15;10(22):2224–34. doi: 10.18632/oncotarget.26776 (PMC6481335; doi:10.18632/oncotarget.26776)
Supplement: Supplementary file 1 [file oncotarget-10-2224-s001.pdf]

The transcriptomic response to irinotecan in colon carcinoma bearing mice preconditioned by fasting

SUPPLEMENTARY MATERIALS

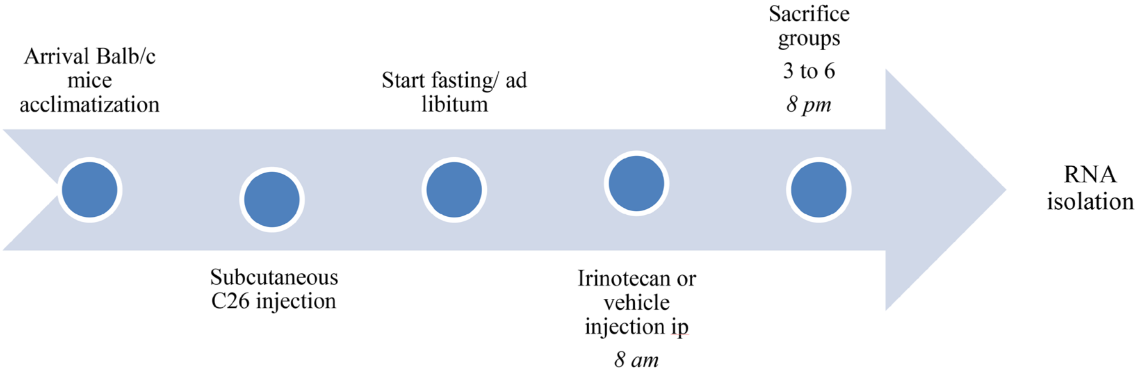

Supplementary Figure 1: Schematic overview of the experimental set-up, including a timeline from arrival of the mice until sacrifice and subsequent RNA isolation for further analyses. Ip = intraperitoneal.

Supplementary Table 1: Regulated upstream transcription factors in liver ranked by their z-score

(A) Liver – AL irinotecan vs. Fasted irinotecan

Upstream regulator	Description	*P*-value	Log ratio	Z-score
**NFE2L2**	Nuclear factor (erythroid-derived 2)-like 2	5.11E-24	−1.477	**+6.267**
**XBP1**	X-box binding protein 1	2.52E-04	−0.197	**+4.531**
**SREBF2**	Sterol regulatory element-binding protein 2	7.03E-11	+0.722	**+4.180**
**SIRT2**	Sirtuin 2	2.02E-06	+0.102	**+3.317**
**PPARGC1B**	Peroxisome proliferator-activated receptor gamma coactivator 1-beta	5.05E-03	−0.176	**+3.197**
**NFE2L1**	Nuclear factor (erythroid-derived 2)-like 1	5.42E-09	+0.300	**+3.162**
**ZBTB20**	Zinc finger and BTB domain-containing protein 20	5.02E-05	+0.205	**+3.153**
**SRF**	Serum response factor	6.16E-03	−0.177	**+3.125**
**SREBF1**	Sterol regulatory element-binding protein 1	1.56E-08	+0.199	**+3.004**
**HSF2**	Heat Shock Transcription Factor 2	4.48E-07	+0.243	**+2.883**
**RB1**	Retinoblastoma 1	1.31E-05	−0.128	**+2.732**
**MRTFB**	Myocardin-related transcription factor B	4.69E-03	+0.298	**+2.714**
**TP73**	Tumor Protein P73	3.01E-02	−0.127	**+2.537**
**SPDEF**	SAM Pointed Domain Containing ETS Transcription Factor	2.77E-02	−0.088	**+2.530**
**IFI16**	Interferon Gamma Inducible Protein 16	1.00E-00	−0.576	**+2.433**
**SOX2**	Sex determining region Y-box 2	1.00E-00	−0.012	**+2.430**
**PPARGC1A**	Peroxisome proliferator-activated receptor gamma coactivator 1-alpha	1.63E-02	−0.965	**+2.430**
**IRF9**	Interferon Regulatory Factor 9	2.74E-01	+0.281	**+2.401**
**KLF15**	Krüppel-like factor 15	7.16E-02	+0.752	**+2.286**
**MED1**	Mediator Complex Subunit 1	1.72E-02	+0.296	**+2.221**
**IRF3**	Interferon Regulatory Factor 3	1.00E-00	+0.184	**+2.171**
**SMAD7**	Mothers Against DPP Homolog 7	1.07E-02	+0.229	**+2.168**
**NFYA**	Nuclear transcription factor Y	8.06E-03	−0.170	**+2.138**
**MYC**	Myc proto-oncogene protein	9.79E-08	−0.354	**+2.122**
**HNF4A**	Hepatocyte nuclear factor 4 alpha	6.09E-41	−0.295	**+2.111**
**MRTFA**	Myocardin-related transcription factor A	1.93E-02	−0.428	**+2.016**
**HAND1**	Heart And Neural Crest Derivatives Expressed 1	3.47E-01	+0.062	**+2.000**
**MYCBP**	C-Myc-binding protein	5.21E-03	+0.370	**+2.000**
**MAFF**	MAF BZIP Transcription Factor F	3.32E-02	−0.494	**+2.000**
**DACH1**	Dachshund Family Transcription Factor 1	1.00E-00	−0.161	**+2.000**
**KDM5A**	Lysine Demethylase 5A	1.07E-04	+0.123	**−4.139**
**NFKBIA**	NFKB Inhibitor Alpha	2.15E-02	−0.325	**−3.762**
**MYB**	MYB Proto-Oncogene	3.54E-01	−0.014	**−3.678**
**STAT3**	Signal transducer and activator of transcription 3	1.33E-01	−0.389	**−3.147**
**GATA6**	GATA Binding Protein 6	3.77E-02	−0.496	**−2.875**
**CREB1**	CAMP responsive element binding protein 1	1.48E-02	−0.256	**−2.617**
**POU5F1**	POU Class 5 Homeobox 1	2.08E-01	+0.071	**−2.607**
**ETS1**	ETS Proto-Oncogene 1	8.36E-02	−0.560	**−2.577**
**KLF3**	Krüppel-like factor 3	1.52E-04	+0.217	**−2.540**
**YAP1**	yes-associated protein 1	3.41E-01	−0.151	**−2.423**
**GATA1**	GATA Binding Protein 1	1.44E-02	+0.087	**−2.338**
**GATA4**	GATA Binding Protein 4	1.00E-00	−0.243	**−2.296**
**CTNNB1**	Catenin Beta 1	2.02E-02	−0.136	**−2.274**
**KLF4**	Krüppel-like factor 4	4.13E-01	+0.608	**−2.263**
**TCL1A**	T Cell Leukemia/Lymphoma 1A	8.23E-05	−0.130	**−2.236**
**AIRE**	Autoimmune regulator	1.00E-00	−0.052	**−2.200**
**SP1**	specificity protein 1	4.52E-04	+0.301	**−2.200**
**NUPR1**	Nuclear Protein 1	4.10E-04	+1.318	**−2.073**
**CEBPD**	CCAAT Enhancer Binding Protein Delta	1.08E-02	−1.214	**−2.034**

Upstream regulator analysis of the DEPS found in the comparison ad libitum irinotecan versus preconditioning by fasting irinotecan in liver. Only z-scores of ≤-2.000 and ≥+2.000 are depicted. Transcription factors are ranked by their z-score.
